# Characterization of the Structural and Mechanical Changes of the Biceps Brachii and Gastrocnemius Muscles in the Subacute and Chronic Stage after Stroke

**DOI:** 10.3390/ijerph20021405

**Published:** 2023-01-12

**Authors:** María Isabel García-Bernal, Paula González-García, Pascal Madeleine, María Jesús Casuso-Holgado, Alberto Marcos Heredia-Rizo

**Affiliations:** 1 Departmento de Fisioterapia, Facultad de Enfermería, Fisioterapia y Podología, Universidad de Sevilla, 41009 Sevilla, Spain; 2 Instituto de Biomedicina de Sevilla (IBIS), 41013 Sevilla, Spain; 3 Sport Sciences—Performance and Technology, Department of Health Science and Technology, Aalborg University, 9220 Aalborg, Denmark; 4 Uncertainty, Mindfulness, Self, Spirituality (UMSS) Research Group, Universidad de Sevilla, 41009 Sevilla, Spain

**Keywords:** stroke, spasticity, muscle, myotonometry, outcomes assessment, ultrasound

## Abstract

The objective of this study was to characterize the changes of muscle tone, stiffness, and thickness of upper and lower limb muscles in stroke survivors. Forty patients with subacute or chronic stroke and 31 controls were included and measured using myotonometry (MyotonPRO), with multiple site assessments at muscle belly (MB) and musculotendinous (MT) locations of the biceps brachii and gastrocnemius muscles. Muscle thickness (ultrasonography) was obtained for each muscle. Upper and lower limb motor performance was evaluated with the Fugl–Meyer Assessment for Upper Extremity and the Functional Ambulance Category. Overall, muscle tone and stiffness were significantly higher at MT than at MB sites. Among stroke patients, differences between the paretic and nonparetic limb were found for the biceps brachii, with lower muscle tone, stiffness, and thickness of the paretic side (all, *p* < 0.05). There were weak to moderate correlations between mechanical (myotonometry) and structural (ultrasound) muscular changes, regardless of the post-stroke stage. This suggests that myotonometry and ultrasonography assess similar, although different, constructs and can be combined in the clinical setting. Their discriminative ability between the paretic and nonparetic sides and between participants with and without stroke differs depending on the muscle, the functional level, and the stroke stage.

## 1. Introduction

Post-stroke spasticity (PSS) is a major sensorimotor disorder that can be present from the early stages after stroke [1] and frequently involves the flexor muscles of the upper limb and the extensor muscles of the lower limb [2]. Structural changes in muscle and tendon appear as a result of PSS, leading to impaired motor control, disuse [3], worse recovery, and disability [4]. However, recent systematic reviews suggest that there is little, and conflicting, evidence about the specific structural and mechanical adaptations that occur in the upper and lower limb muscles after stroke [5,6]. Understanding this could help to guide clinical decisions aimed at targeting focal spasticity of specific muscles and locations within the muscles (i.e., botulin toxin) [7] or at restoring muscle function during physical rehabilitation [8].

Monitoring structural and mechanical soft-tissue properties in PSS is a complex task. Clinical measures are indirect, suboptimal, and show limited reliability and reproducibility [9]. Alternatively, direct measurement tools, such as ultrasound elastography and myotonometry, have emerged as promising procedures to provide quantitative useful information in patient populations [10,11,12]. Current literature suggests a non-uniform distribution of the muscle mechanical properties after stroke, with different adaptations in the upper and lower limbs [8,13]. However, it has been recommended to measure several spots within the muscle and tendon to better characterize PSS [13]. Topographical mapping, based on multiple site assessments within an area of interest, has been used extensively for surface electromyography (SEMG) [14] and quantitative sensory testing [15] and, more recently, to report the spatial distribution of muscle stiffness in people with chronic pain [16].

The objective of this study was to characterize the topographical maps of tone and stiffness of the biceps brachii and gastrocnemius muscles in stroke patients, compared with healthy controls, and observe the possible association between structural (muscle thickness) and mechanical (muscle tone and stiffness) changes. We hypothesized that: (1) among persons with stroke, musculotendinous sites would show higher tone and stiffness than muscle belly sites, structural and mechanical changes would be correlated, and muscle adaptations would differ between the upper and the lower limb; (2) myotonometry and ultrasound measurements would be able to discriminate between participants with and without stroke.

## 2. Materials and Methods

### 2.1. Design

This cross-sectional study was approved by the Institutional Review Board (Junta de Andalucía Ethical Committee for Biomedical Research, code number CI 1222-N-16) and followed the STROBE guidelines for observational studies. All participants provided written informed consent prior to data collection.

### 2.2. Participants

This is a secondary analysis. The sample was the same as in a previous study investigating the influence of muscle position during assessment (relaxed vs. stretched) on the mechanical properties of spastic muscles [13]. Stroke participants were recruited from public and private settings and assigned to either group according to time after stroke. Twenty stroke patients in the subacute stage (1.5 to 9 months after the event) and 20 patients in the chronic stage (more than 9 months after the event) [17] participated in the study. A total of 31 healthy participants, from the same population-based cohort, were included in the control group. Patients with a first-ever stroke were selected if they had a score ≥ 1 in the Modified Ashworth Scale for the biceps brachii and gastrocnemius muscles [18] and showed a normal cognitive status (score ≥ 24 in the Mini-Mental State Examination) [19]. The exclusion criteria were: severe upper or lower limb injury, a diagnosed behavioral disorder, recent changes in medication for PSS, having received botulinum toxin injections within 3 months, and any other neurological disease. Stroke participants were undergoing regular care, mostly involving physical therapy and medication intake.

### 2.3. Outcome Measures

**Figure 1:**
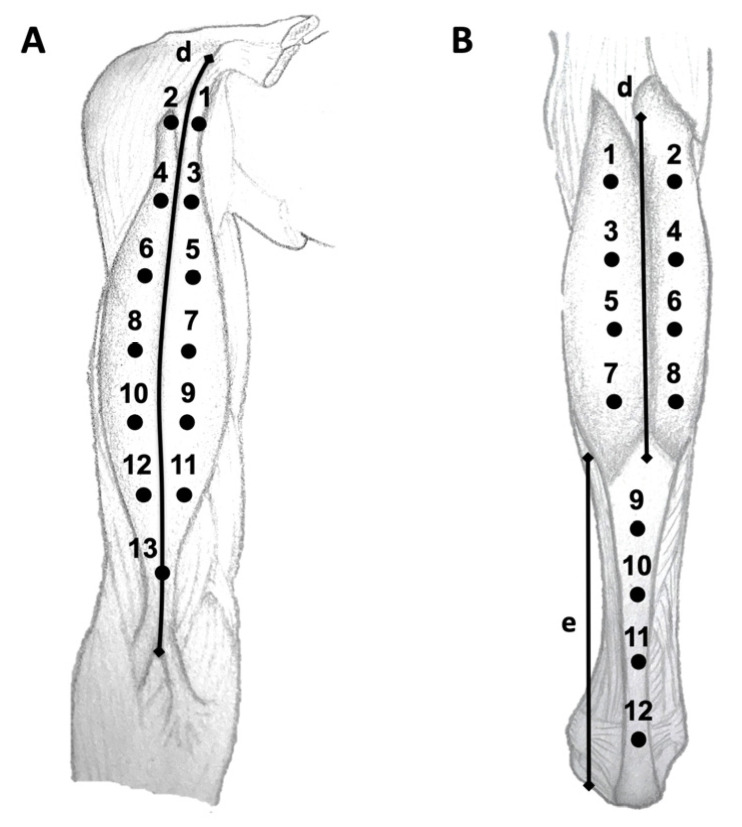
Measurement sites for the biceps brachii (**A**) and gastrocnemius (**B**) muscles.

Tone and stiffness of the biceps brachii and gastrocnemius muscles were measured with the MyotonPRO (Myoton AS, Tallinn, Estonia), using several site assessments. The MyotonPRO is a noninvasive hand-held device that applies consecutive short mechanical impulses on the surface of the skin to induce a dynamic soft-tissue response (Figure S1). Using an accelerometer, muscle tone (tension) is measured as the frequency of the signal and muscle stiffness is the resultant natural oscillation that characterizes the resistance to the external force [20]. For the biceps brachii, participants lay supine, with 45° elbow flexion and neutral forearm. First, we measured the distance d between the coracoid process of the scapula and the muscle insertion at the radial tuberosity. Then, using a wax pencil, a 13-point grid was drawn, with adjacent points separated longitudinally by 1/8 of the d value and transversally by 1/6 of d, except for point 13, which was separated by 1/12 of d. Points 1, 2, 3, 4 and 13 corresponded to musculotendinous (MT) sites, whereas points 5, 6, 7, 8, 9, 10, 11, and 12 were located at the muscle belly (MB). For the gastrocnemius, participants were prone, with the knee in 45° flexion. We calculated the distance d between the upper edge of the calcaneus and the lower edge of the gastrocnemius medialis, and e between the lower edge of the gastrocnemius medialis and the midpoint between the medial and lateral condyle of the femur. Points 1 to 8, corresponding to MB sites, were separated longitudinally by 1/5 of e and transversally by 1/11 of e. Points 9 to 12 were located at MT sites, with a distance between adjacent points of 1/5 of d. Assessment sites are described in detail in Figure 1. All measures were taken twice for each site bilaterally, and the average score was used for analysis [16]. The topographical maps were generated with the Matlab software 9.1 (The Mathworks, Natick, MA, USA). For that purpose, muscle tone and stiffness data from the MyotonPRO were used, applying an inverse distance weighted interpolation method.

Resting measures of muscle thickness were obtained bilaterally at points 7 and 8 for the biceps brachii and at points 5 and 6 for the gastrocnemius muscle. We used B-mode ultrasound imaging (4.2 to 13 MHz) coupled with a multifrequency linear array transducer, model 12L-RS (GE Logiq V2, GE Health Care, Chicago, IL, USA). The average of two consecutive measures was taken for analysis. The muscle thickness was measured as the vertical distance between the superficial and deep aponeurosis for the gastrocnemius and between the adipose tissue–muscle interface and the muscle–bone interface for the biceps brachii [21].

Upper and lower limb motor performance was evaluated with the Fugl–Meyer Assessment for Upper Extremities (FMA-UE) [22] and the Functional Ambulation Category (FAC) [23], respectively. The same examiners, who remained blinded to participants’ allocation group, collected all demographic and clinical measures.

### 2.4. Statistical Analysis

Sample size was estimated using the software G*Power (v. 3.1.9.2, Heinrich-Heine University, Düsseldorf, Germany). We considered an alpha level of 0.05, an 80% power, and a large effect size (*n*^2^ = 0.15) for differences in muscle tone and stiffness between groups. This analysis revealed that at least 19 participants were required per study group.

The PASW Advanced Statistics (SPSS Inc., Chicago, IL, USA), version 26.0, was used for statistical processing. Normal distribution of the data was analyzed with the Shapiro–Wilk test. A three-way analysis of variance (ANOVA) was used to detect differences in tone and stiffness of the biceps brachii and gastrocnemius, using sites (MT vs. MB) and sides (paretic vs. nonparetic) of testing as the within-participant factors, and group as the between-participant factor. Associations between muscle thickness and myotonometry were tested with the Pearson product-moment correlation coefficient analysis or the Spearman rank test. The level of significance was set to *p* < 0.05.

## 3. Results

**Figure 2:**
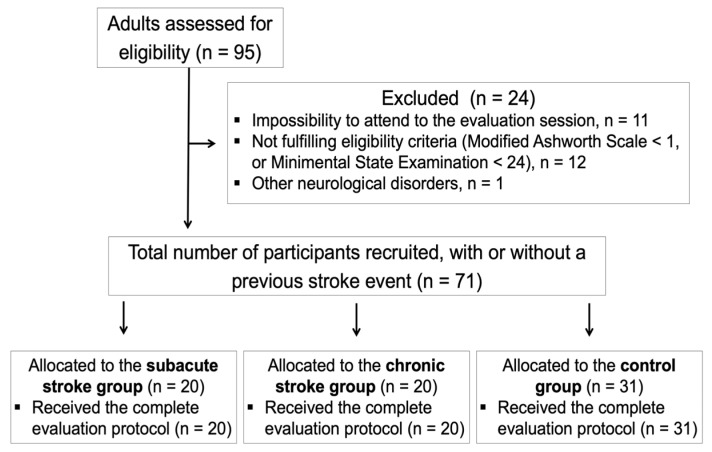
Flowchart diagram of the study participants.

A total of 71 adults (31 controls, 20 subacute stroke, and 20 chronic stroke) were included (Figure 2).

The baseline clinical and demographic features of participants can be seen in Table 1. Upper limb motor performance was severely compromised among stroke survivors (overall score, 19 ± 20.8 points), whereas 15% of them (6/30) could be categorized as nonfunctional ambulators, according to the FAC (FAC = 0).

**Figure 3:**
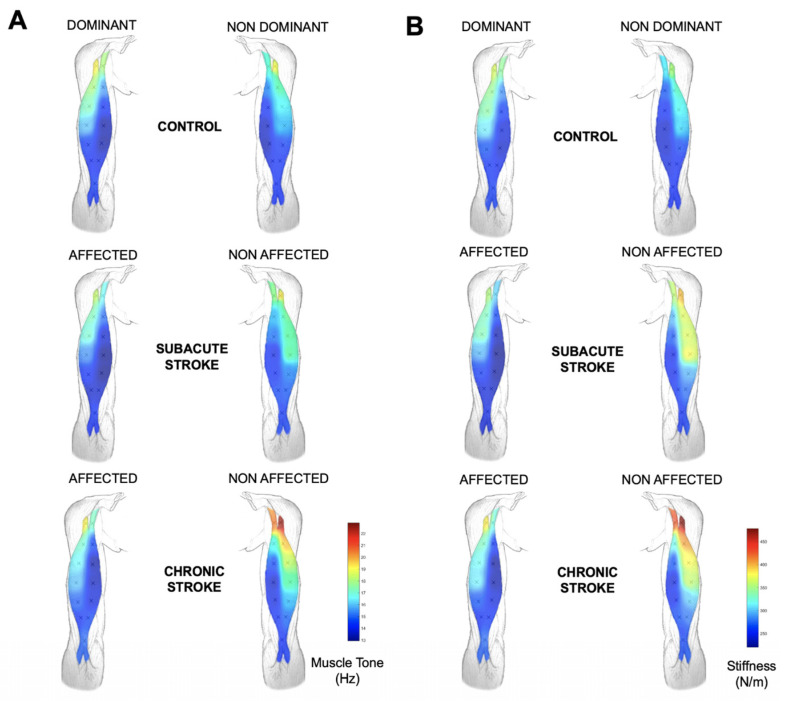
Muscle tone (Hz; **A**) and stiffness (N/m; **B**) maps based on average values of the assessed locations from the biceps brachii muscle in individuals with subacute and chronic stroke, and control participants.

**Figure 4:**
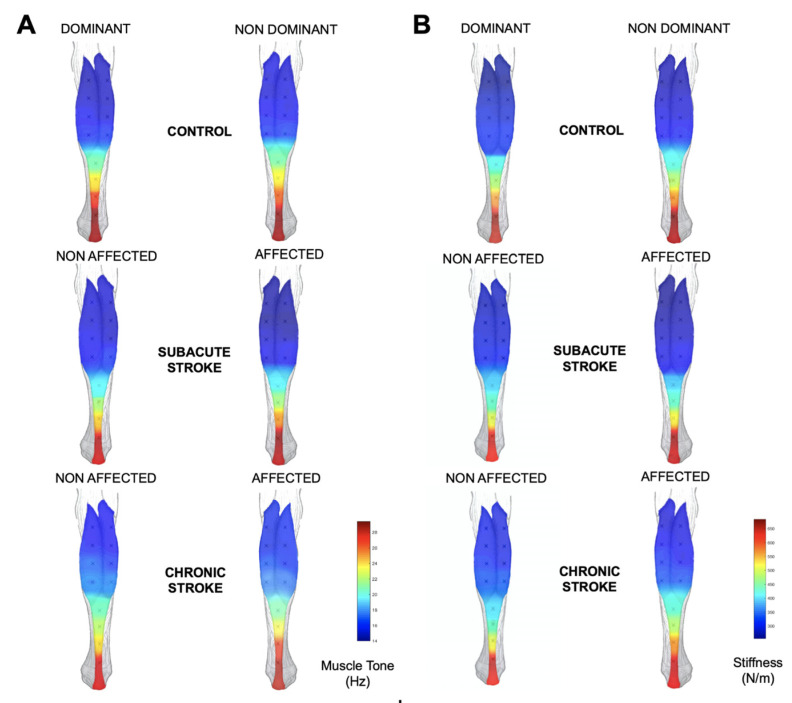
Muscle tone (Hz; **A**) and stiffness (N/m; **B**) maps based on average values of the assessed locations from the gastrocnemius muscle in individuals with subacute and chronic stroke, and control participants.

The topographical maps of tone and stiffness of the biceps brachii and gastrocnemius muscles are shown in Figure 3 and Figure 4.

Table 2 and Table 3 include the mean ± SD values for tone and stiffness at the different sites, sides, and groups. Table 4 shows the data for muscle thickness.

### 3.1. Mechanical and Structural Muscular Adaptations among Stroke Survivors

For the biceps brachii, the ANOVA revealed a significant sites*sides interaction for muscle tone (F = 1.937; *p* = 0.020; η^2^ = 0.023) and stiffness (F = 2.728; *p* = 0.001; η^2^ = 0.032), and a side effect for muscle thickness (F = 19.367; *p* < 0.001; η^2^ = 0.110). For the gastrocnemius muscle, a sides*group interaction was found for stiffness (F = 4.269; *p* = 0.039; η^2^ = 0.004). Tone and stiffness were significantly higher at MT than at MB sites, and in patients in the chronic stage compared with the subacute stroke group (all, *p* < 0.001). There were differences between sides for the biceps brachii, with lower tone, stiffness, and thickness of the affected upper limb (*p* < 0.001). Muscle thickness was correlated with tone (r = 0.355, *p* < 0.001) and stiffness (r = 0.353, *p* < 0.001) for the biceps brachii, and with stiffness for the gastrocnemius (r = 0.237, *p* = 0.003).

### 3.2. Discriminative Ability between Stroke Survivors and Healthy Controls

A significant group*site interaction was demonstrated for the biceps brachii stiffness (F = 1.732; *p* = 0.010; η^2^ = 0.023) and for the gastrocnemius muscle tone (F = 1.942; *p* = 0.003; η^2^ = 0.025) and stiffness (F = 1.742; *p* = 0.012; η^2^ = 0.023). Muscle tone and stiffness were higher in chronic stroke patients than in controls, and lower in the subacute stroke group compared with healthy participants (only for the gastrocnemius). Stroke survivors showed lower biceps brachii and gastrocnemius muscle thickness than did those in the control group (all, *p* < 0.05).

## 4. Discussion

The structural and mechanical properties of the biceps brachii and gastrocnemius muscles were heterogeneously distributed among stroke survivors. Tone and stiffness were higher at MT than at MB sites, and the biceps brachii muscle tone, stiffness, and thickness were lower at the affected side. Myotonometry and ultrasound measures were significantly correlated, and both techniques could discriminate between the paretic and nonparetic upper limb and between participants with and without stroke.

### 4.1. Mechanical and Structural Muscular Adaptations among Stroke Survivors

Current literature has characterized the muscle adaptations after stroke with SEMG [14], mechanomyography [24], and ultrasonography [6,8]. Myotonometry uses superficial mechanical deformation and represents a convenient approach that is considerably less costly than elastography [25] and has good psychometric properties [11]. Hence, it can be of high clinical value to track muscle mechanical changes [26], especially in stroke populations [9]. Previous research with myotonometry has measured a single site, often at the midportion of the MB, which does not reflect the spatial distribution [11]. This is the first study in which myotonometry is used to quantify multiple sites within the MB and tendon to image the differences in tone and stiffness between upper and lower limb muscles in people with subacute or chronic stroke.

In both biceps brachii and gastrocnemius muscles, tone and stiffness were significantly higher at MT than at MB sites, in line with findings in adults with spinal cord injury [27] and Parkinson’s disease [28]. These differences can be explained by the structural and functional adaptations that occur after stroke, such as variations in the number and length of sarcomeres in the skeletal muscles [29]. Additionally, reduced muscle thickness [6], together with increased tendon compliance and muscle pennation angle [30], leads to muscle atrophy, which is usually observed following prolonged disuse. In fact, disuse is considered the main factor involved in most of these muscular changes [6].

Differences in the level of function may help to understand the distinct behaviors of the upper and lower limb muscles. Participants with stroke reported a severe upper limb motor impairment (FMA-UE < 19) that denotes a restricted ability to function (e.g., bring the arm into the body, extend the elbow, or relax the fingers) [31]. The involvement of the paretic upper limb in daily activities is related to the extent of motor restriction [32]. The most natural way to respond to that is to rely on the nonaffected upper limb [33]. This would explain the decreased tone, stiffness, and thickness of the biceps brachii of the affected side. As regards the lower limb motor performance, 45% of stroke participants (18/40) were able to walk without physical assistance (FAC ≥ 3) [23]. Despite the fact that their walking ability was somehow preserved, stroke survivors tend to be inactive and sedentary [34]. Several studies conclude that changes in muscle architecture after stroke may not be limited to the spastic side [35], and that the nonparetic lower limb also adapts [5,6]. For example, the bilateral overuse of lower limb muscles to walk or support body weight [36] may account for the lack of differences between sides for the gastrocnemius muscle tone, stiffness, and thickness. Overall, some of the adaptations of the lower limb have been described as muscle specific [37], which makes it difficult to reach a definite conclusion. These findings seem to suggest the need to involve both lower extremities during rehabilitation, although further research is necessary to support this claim.

Weak to moderate correlations were observed between myotonometry and ultrasound measures [21,36,38], with inconsistent results across muscles. According to these findings, both techniques assess similar, although different, constructs and, therefore, can be combined for the clinical assessment of PSS [21].

### 4.2. Discriminative Ability between Stroke Survivors and Healthy Controls

Changes in muscle architecture can take place as early as 3 weeks after stroke [39]. Muscle thickness decreases in the first months of recovery [40], whereas the evolution to the chronic stage leads to increased muscle tone and stiffness. This has been shown in the upper [41,42] and lower [36,38] limb in chronic stroke survivors. Neural and morphological changes can support these findings [43]. However, evidence is still conflicting about how the structural and mechanical muscle properties may evolve with time. For example, Mirbagheri et al. described two temporal patterns of change for muscle stiffness over the first 12 months: a progressive increase or a slow decline after the first four weeks in patients with mild motor impairment [44]. Again, the level of motor performance represents a key aspect in most of these changes. The current results indicate that treatment strategies must be carefully chosen according to the stroke stage. Additional research may help to understand the best therapeutic approach at each stage. Previous studies concluded that task-oriented repetitive training can be recommended during the acute and subacute stages to speed recovery and prevent disuse [45]. For the chronic stage, multimodal rehabilitation programs including localized soft-tissue therapies may be of more interest. Longitudinal studies monitoring the structural and mechanical muscular changes in larger cohorts are warranted to improve the clinical management of PSS.

### 4.3. Study Limitations

Chronic stage was defined as more than 9 months post-stroke [17], although new standards may recommend otherwise. Assessments were conducted in the relaxed position to prevent fatigue. There is a high heterogeneity among studies in the measurement protocols using myotonometry and ultrasonography, which may negatively impact the clinical interpretation of the results [11,21]. Future studies could also combine SEMG and mechanomyography as an index of muscle performance with myotonometry or ultrasonography, as recently performed in healthy individuals [46]. Stroke patients were involved in different treatment routines. Since physical activity and medication intake can modulate muscle tone, the possible impact of the physical rehabilitation programs on the results needs to be considered.

## 5. Conclusions

Topographical maps of the biceps brachii and gastrocnemius muscles revealed a heterogeneous distribution of structural and mechanical properties, with lower muscle tone, stiffness, and thickness of the paretic upper limb, and increasing tone and stiffness in MT compared with MB locations and from subacute to chronic stroke stage. Among stroke survivors, the discriminative ability of myotonometry and ultrasonography can be influenced by the assessed muscle, the stroke stage, and the level of motor performance.

## Figures and Tables

**Table 1:** Baseline clinical and demographic features of participants.

	Subacute Stroke (*n* = 20)	Chronic Stroke (*n* = 20)	Control Group (*n* = 31)	*p* Value
Age (years)	60.2 ± 9.7	61.45 ± 9.7	60.8 ± 10.6	0.926
Sex, female, % (*n*)	35% (7)	35% (7)	45.2% (14)	0.689
Time after stroke (weeks)	17 (6–34)	242.5 (58–1108)	N/A	<0.001
Affected side, left, % (*n*)	55% (11)	75% (15)	N/A	0.289
Hand dominance, right, left, ambidextrous, % (*n*)	100% (20)	85% (17); 5% (1); 10% (2)	80.6% (25); 19.4% (6)	0.131
Leg dominance, right, left, ambidextrous, % (*n*)	95% (19); 5% (1);	80% (16); 5% (1); 15% (3)	83.9% (26); 16.1% (5)	0.319

**Table 2:** Mean ± SD biceps brachii stiffness (N/m) and tone (Hz) over assessed sites (points 1 to 13, MB and MT sites) in stroke patients and controls.

		Control Participants (*n* = 31)		Subacute Stroke (*n* = 20)		Chronic Stroke (*n* = 20)	
Site	Measure	Dominant Side	Non-Dominant Side	Affected Side	Non-Affected Side	Affected Side	Non-Affected Side
Point 1	Stiffness Tone	311.1 ± 82.7 17.2 ± 2.7	343.9 ± 78.7 18.2 ± 2.5	302.9 ± 101.9 16.7 ± 3.1	355.5 ± 112.8 18.1 ± 3.4	322.4 ± 94.3 17.3 ± 3.0	434.1 ± 139.2 20.6 ± 3.9
Point 2	Stiffness Tone	359.5 ± 55.9 19.0 ± 2.4	372.9 ± 58.1 19.3 ± 2.3	357.7 ± 59.8 18.5 ± 2.2	400.5 ± 97.1 19.5 ± 4.1	388.9 ± 85.6 19.5 ± 3.5	478.2 ± 111.0 22.9 ± 4.2
Point 3	Stiffness Tone	264.9 ± 49.8 15.1 ± 2.0	283.4 ± 49.1 15.6 ± 1.9	260.9 ± 48.0 15 ± 2.0	293.4 ± 71.9 15.7 ± 2.5	254.6 ± 46.4 14.5 ± 1.9	315.7 ± 61.1 16.7 ± 2.2
Point 4	Stiffness Tone	335.7 ± 52.7 17.6 ± 2.2	346.5 ± 43.2 18.0 ± 1.7	335.7 ± 55.3 17.3 ± 2.6	370.8 ± 69.8 17.9 ± 3.2	332.5 ± 51.5 17.1 ± 2.6	407.4 ± 85.4 19.8 ± 3.2
Point 5	Stiffness Tone	250.5 ± 47.7 14.1 ± 2.0	256.9 ± 52.2 14.2 ± 1.9	227.4 ± 41.3 13.5 ± 1.6	267.3 ± 63.3 15.0 ± 2.5	223.0 ± 47.2 13.2 ± 2.0	265.6 ± 67.1 14.5 ± 2.6
Point 6	Stiffness Tone	319.1 ± 49.7 16.6 ± 2.2	340.6 ± 57.6 17.4 ± 2.3	339.7 ± 82.9 17.1 ± 3.2	369.7 ± 89.3 17.8 ± 3.4	318.5 ± 67.6 16.6 ± 2.9	380.1 ± 96.0 18.6 ± 3.5
Point 7	Stiffness Tone	235.2 ± 26.4 13.4 ± 1.6	240.2 ± 29.8 13.2 ± 1.4	220.4 ± 35.6 13.0 ± 1.5	242.9 ± 30.7 13.7 ± 1.5	228.0 ± 29.7 13.5 ± 1.3	238.9 ± 31.8 13.4 ± 1.3
Point 8	Stiffness Tone	307.2 ± 78.7 15.9 ± 2.9	312.4 ± 67.5 16.1 ± 2.5	297.8 ± 75.5 15.5 ± 2.5	381.6 ± 131.3 17.8 ± 4.0	315.3 ± 70.4 16.1 ± 2.2	364.1 ± 101.2 17.7 ± 3.4
Point 9	Stiffness Tone	237.3 ± 23.9 13.3 ± 1.3	235.3 ± 19.8 13.1 ± 1.0	223.9 ± 31.4 12.9 ± 1.6	245.4 ± 29.1 14.1 ± 1.9	242.6 ± 31.9 13.7 ± 1.5	245.3 ± 23.7 13.7 ± 1.2
Point 10	Stiffness Tone	262.1 ± 38.9 14.4 ± 1.8	264.9 ± 37.1 14.4 ± 1.9	260.1 ± 41.5 14.2 ± 1.6	294.1 ± 47.4 15.2 ± 1.9	267.9 ± 30.7 14.9 ± 1.5	302.2 ± 44.1 15.9 ± 2.1
Point 11	Stiffness Tone	242.5 ± 20.8 13.7 ± 1.0	246.9 ± 26.7 13.9 ± 1.2	233.9 ± 26.7 13.6 ± 1.5	251.8 ± 37.8 14.5 ± 1.8	257.2 ± 32.6 14.6 ± 1.9	263.3 ± 25.7 14.2 ± 1.6
Point 12	Stiffness Tone	247.9 ± 44.9 14.2 ± 1.8	252.9 ± 37.8 14.0 ± 1.6	243.3 ± 32.5 13.5 ± 1.2	276.1 ± 43.1 14.8 ± 1.6	248.2 ± 23.2 14.1 ± 1.4	280.3 ± 37.3 14.9 ± 1.9
Point 13	Stiffness Tone	253.4 ± 39.7 14.3 ± 1.4	258.8 ± 48.4 14.5 ± 1.9	245.6 ± 33.2 14.3 ± 1.3	251.9 ± 54.2 14.4 ± 2.5	275.7 ± 75.2 15.1 ± 2.9	259.9 ± 39.1 14.2 ± 1.7
Muscle belly (MB) sites	Stiffness Tone	256.8 ± 27.1 14.3 ± 1.4	262.2 ± 28.1 14.3 ± 1.3	248.1 ± 36.3 13.9 ± 1.3	277.7 ± 35.8 15.1 ± 1.5	254.8 ± 27.6 14.4 ± 1.2	280.9 ± 35.4 15.0 ± 1.5
Musculotendinous (MT) sites	Stiffness Tone	295.3 ± 41.2 16.4 ± 1.7	310.2 ± 36.0 16.8 ± 1.5	288.7 ± 37.8 16.1 ± 1.5	318.1 ± 58.1 16.7 ± 2.3	302.4 ± 49.9 16.4 ± 2.1	351.9 ± 50.7 18.1 ± 2.1

**Table 3:** Mean ± SD gastrocnemius muscle stiffness (N/m) and tone (Hz) over assessed sites (points 1 to 12, MB and MT sites) in stroke patients and controls.

		Control Participants (*n* = 31)		Subacute Stroke (*n* = 20)		Chronic Stroke (*n* = 20)	
Site	Measure	Dominant Side	Non-Dominant Side	Affected Side	Non- Affected Side	Affected Side	Non-Affected Side
Point 1	Stiffness Tone	255.4 ± 22.1 14.6 ± 1.1	271.5 ± 37.6 15.3 ± 1.8	253.9 ± 29.6 14.4 ± 1.5	273.5 ± 47.4 15.2 ± 2.3	283.4 ± 52.5 15.4 ± 2.2	295.1 ± 65.8 15.8 ± 2.5
Point 2	Stiffness Tone	259.3 ± 28.1 14.9 ± 1.2	267.2 ± 33.1 15.2 ± 1.4	263.1 ± 39.2 14.5 ± 1.8	273.3 ± 51.6 14.9 ± 2.4	292.8 ± 43.3 15.8 ± 1.8	291.5 ± 73.4 15.7 ± 2.8
Point 3	Stiffness Tone	277.5 ± 24.8 14.9 ± 1.4	280.7 ± 23.2 15.0 ± 1.3	265.0 ± 25.2 14.2 ± 1.4	282.8 ± 40.5 15.0 ± 2.2	298.1 ± 43.3 15.4 ± 2.5	300.8 ± 59.4 15.9 ± 2.6
Point 4	Stiffness Tone	279.1 ± 19.0 14.7 ± 1.4	283.4 ± 26.4 15.3 ± 1.6	269.9 ± 19.6 14.0 ± 1.3	281.4 ± 30.8 14.8 ± 1.8	289.9 ± 40.7 15.6 ± 2.3	290.7 ± 66.2 15.6 ± 2.9
Point 5	Stiffness Tone	288.8 ± 26.6 15.7 ± 1.4	290.7 ± 28.1 15.9 ± 1.5	278.7 ± 31.3 14.9 ± 1.8	289.3 ± 29.7 15.7 ± 2.0	317.4 ± 51.7 16.8 ± 2.8	312.9 ± 58.4 17.1 ± 3.0
Point 6	Stiffness Tone	282.3 ± 18.7 14.9 ± 1.5	281.6 ± 26.2 15.3 ± 1.7	278.0 ± 26.7 14.4 ± 1.7	284.3 ± 30.2 14.8 ± 2.0	293.5 ± 40.7 15.8 ± 2.3	295.7 ± 66.5 16.2 ± 2.6
Point 7	Stiffness Tone	306.8 ± 26.3 16.0 ± 1.4	308.1 ± 25.4 16.2 ± 1.5	290.4 ± 25.1 15.3 ± 1.5	299.2 ± 31.7 15.7 ± 2.2	334.0 ± 58.3 17.9 ± 3.1	327.0 ± 66.2 17.7 ± 2.8
Point 8	Stiffness Tone	314.7 ± 29.2 16.2 ± 1.7	310.7 ± 27.9 16.5 ± 1.6	305.4 ± 26.8 15.7 ± 1.8	308.8 ± 37.3 16.4 ± 2.5	332.3 ± 47.1 17.5 ± 2.7	329.4 ± 57.2 17.6 ± 3.2
Point 9	Stiffness Tone	416.4 ± 40.4 21.1 ± 2.2	421.6 ± 40.9 21.1 ± 2.2	380.6 ± 49.1 19.3 ± 2.3	386.9 ± 44.5 19.7 ± 2.4	428.9 ± 81.9 20.9 ± 3.2	400.8 ± 71.4 20.8 ± 3.1
Point 10	Stiffness Tone	483.2 ± 58.6 23.9 ± 3.3	482.5 ± 56.5 23.5 ± 2.6	435.3 ± 59.8 21.8 ± 2.7	440.2 ± 60.8 22.0 ± 2.5	482.3 ± 89.5 23.4 ± 3.6	445.7 ± 77.6 22.4 ± 3.5
Point 11	Stiffness Tone	562.1 ± 90.1 26.9 ± 3.9	571.3 ± 98.3 26.3 ± 3.6	521.7 ± 98.9 25.2 ± 3.9	509.5 ± 66.6 24.4 ± 3.1	572.1 ± 117.5 26.8 ± 4.5	510.4 ± 98.2 24.0 ± 4.0
Point 12	Stiffness Tone	679.4 ± 91.1 29.3 ± 2.8	683.8 ± 119.5 29.4 ± 3.8	670.1 ± 164.1 29.2 ± 4.6	663.8 ± 109.8 28.6 ± 3.5	651.4 ± 121.5 28.4 ± 3.5	663.6 ± 164.9 28.2 ± 5.0
Muscle belly (MB) sites	Stiffness Tone	280.3 ± 13.7 15.1 ± 0.9	284.2 ± 17.4 15.5 ± 1.1	273.3 ± 19.8 14.6 ± 1.0	284.0 ± 29.4 15.2 ± 1.7	301.3 ± 35.5 16.0 ± 1.9	302.3 ± 61.1 16.3 ± 2.5
Musculotendinous (MT) sites	Stiffness Tone	515.9 ± 56.1 24.8 ± 2.4	475.2 ± 55.3 23.1 ± 2.4	476.4 ± 70.5 23.2 ± 2.8	478.2 ± 55.4 23.1 ± 2.3	518.1 ± 98.4 24.4 ± 3.4	484.2 ± 84.2 23.4 ± 3.3

**Table 4:** Muscle thickness (cm) of the biceps brachii and gastrocnemius muscles in stroke survivors.

		Subacute Stroke (*n* = 20)		Chronic Stroke (*n* = 20)		Control Group (*n* = 31)	
Muscle	Site	Affected Side	Non-Affected Side	Affected Side	Non-Affected Side	Dominant Side	Non-Dominant Side
Biceps brachii	Point 7 Point 8	1.97 ± 0.52 1.41 ± 0.36	2.27 ± 0.66 1.78 ± 0.59	1.83 ± 0.39 1.24 ± 0.28	2.33 ± 0.45 1.66 ± 0.51	2.41 ± 0.51 1.75 ± 0.72	2.37 ± 0.59 1.57 ± 0.48
Gastroc-nemius	Point 5 Point 6	1.47 ± 0.42 1.34 ± 0.45	1.60 ± 0.26 1.29 ± 0.46	1.25 ± 0.51 1.12 ± 0.41	1.45 ± 0.47 1.35 ± 0.44	1.70 ± 0.41 1.41 ± 0.29	1.75 ± 0.40 1.41 ± 0.28

## Data Availability

The data presented in this study are available on request from the corresponding author.

## Supplementary Materials

The following supporting information can be downloaded at: https://www.mdpi.com/article/10.3390/ijerph20021405/s1, Figure S1: MyotonPRO device in use.
