# Supplementary figures and images for: Characterization of the Structural and Mechanical Changes of the Biceps Brachii and Gastrocnemius Muscles in the Subacute and Chronic Stage after Stroke
Source: Int J Environ Res Public Health. 2023 Jan 12;20(2):1405. doi: 10.3390/ijerph20021405 (PMC9864550; doi:10.3390/ijerph20021405)

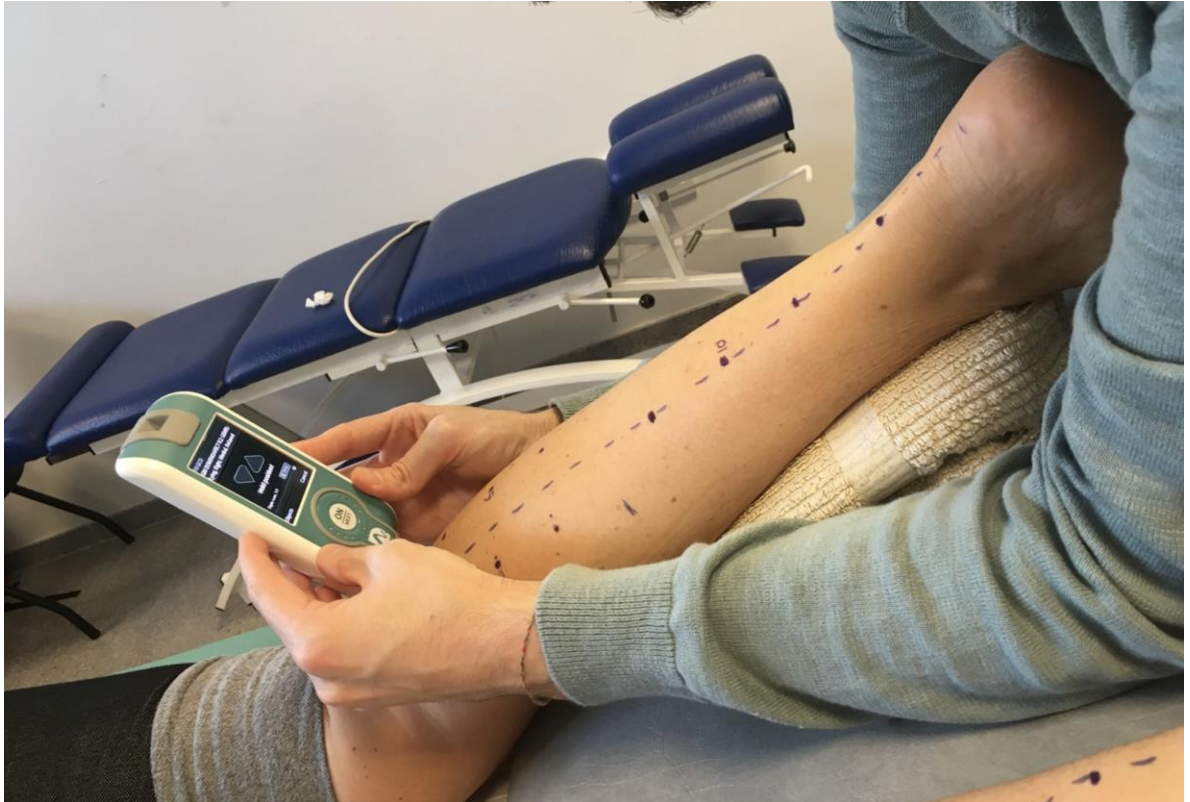

**Figure S1:** MyotonPRO device in use.

Supplement: Supplementary file 1 [file ijerph-20-01405-s001.zip › ijerph-2080175-supplementary.pdf]
